# Burden of Illness in Prostate Cancer Patients with a Low-to-Moderate Risk of Progression: A One-Year, Pan-European Observational Study

**DOI:** 10.1155/2014/472949

**Published:** 2014-03-13

**Authors:** Cesare Selli, Anders Bjartell, Javier Burgos, Matthew Somerville, Juan-Manuel Palacios, Laure Benjamin, Libby Black, Ramiro Castro

**Affiliations:** ^1^Department of Urology, University of Pisa, 56126 Pisa, Italy; ^2^Skåne University Hospital, SE 205 02 Malmö, Sweden; ^3^Hospital Ramon y Cajal, 28034 Madrid, Spain; ^4^GlaxoSmithKline, Research Triangle Park, NC 27709, USA; ^5^GlaxoSmithKline, Urology Centre of Excellence, C/Severo Ochoa 2, Tres Cantos, 28760 Madrid, Spain; ^6^GlaxoSmithKline, Health Outcomes Studies, 78160 Marly-Le-Roi, France; ^7^GlaxoSmithKline, King of Prussia, PA 19406, USA

## Abstract

*Objective*. To assess the impact of low-to-moderate risk prostate cancer on patients' quality of life (QoL) at diagnosis and within the first year of treatment. *Subjects and Methods*. Men (*n* = 672) aged 50–75 years with prostate cancer (Gleason score ≤7, PSA ≤20 ng/mL and clinical staging T1c–T2b) were enrolled in five European countries. Patients completed five questionnaires, including EORTC Quality of Life Questionnaire—Prostate Cancer 25 (QLQ-PR25) and EORTC Quality of Life Questionnaire—Cancer 30 (QLQ-C30). Questionnaires were completed at baseline, at 3 months and 12 months after starting treatment. The primary endpoint was the change in QLQ-PR25 urinary symptoms subscale score from baseline to the assessment at 3 months. *Results*. Mean (SD) age was 65.0 (5.7) years and 400 (66%) men had Gleason score ≤6 prostate cancer. The most frequently used initial treatment was radical prostatectomy (71% of patients). QLQ-PR25 urinary symptoms subscale score was significantly increased at 3 months (*P* < 0.001), indicating that urinary symptoms worsened after treatment. The score was lower at 12 months than at 3 months, but it was still significantly higher than at baseline (*P* < 0.001). Hormonal treatment-related symptoms, sexual functioning, and sexual activity scores significantly worsened at 3 and 12 months (all *P* < 0.001). For the QLQ-C30 questionnaire, global health status/QoL score significantly decreased at month 3 but was not different from baseline by month 12. Scales for physical, role, and social functioning, and fatigue, showed significant deterioration at 3 and 12 months. *Conclusions*. Low-to-moderate risk prostate cancer may have a substantial effect on patients' QoL within one year following treatment.

## 1. Introduction

In 2008, the estimated number of new prostate cancer cases worldwide was almost 900,000; this burden is expected to increase to 1.7 million by 2030 due to the growth and aging of the global population [1]. Prostate cancer was the most frequently diagnosed male cancer (excluding skin cancer) in Europe in 2008 with an estimated 382,000 cases or 22% of all male cancers diagnosed [2]. Furthermore, it is the third most common cause of cancer death in men in Europe after lung and colorectal cancer, with over 89,000 deaths attributed to prostate cancer in 2008.

The burden associated with prostate cancer diagnosis is high and stems from the diagnosis, the disease itself, and the varying impact of the available treatment options. The majority (90%) of men with low-risk prostate cancers receive radical intervention, with 50–60% undergoing radical prostatectomy as their primary treatment [3, 4]. Side effects such as sexual dysfunction, urinary incontinence, bowel problems, anxiety, weakness, fatigue, hot flushes, and pain are frequently experienced, depending on the type of treatment given [5–9]. Existing data also confirm the impact of different prostate cancer treatments, or the disease in general, on quality of life (QoL) and patients' emotional well-being [6, 7]. However, there is little (if any) information available on the burden of illness in men diagnosed with low-to-moderate risk prostate cancer. A recent study assessed the long-term (5-year follow-up) QoL impact of treatments for low or intermediate risk prostate cancer in 704 patients [10]. Men were treated with radical prostatectomy, external beam radiotherapy, or brachytherapy, with brachytherapy shown to cause the least impact on QoL. The present pan-European study assessed the shorter-term impact of low-to-moderate risk prostate cancer on patients' QoL and anxiety/depression (i.e., at diagnosis and within the first year of treatment) and estimated healthcare consumption within the first year of diagnosis.

## 2. Subjects and Methods

This was a prospective, 1-year, observational, pan-European (Germany, France, Spain, Italy, and Sweden) study of men aged 50–75 years with prostate cancer of low-to-moderate risk of progression (Gleason score ≤ 7, PSA ≤ 20 ng/mL and clinical staging T1c–T2b according to D'Amico criteria of low-intermediate risk [11]). All included patients were able to read and write in order to complete the study questionnaires. Exclusion criteria comprised the following: Gleason score ≥ 8, PSA > 20 ng/mL, or clinical staging ≥ T2c; previous treatment for prostate cancer or use of prostate cancer-related medications; the presence of any other cancer (except basal cell carcinoma) within the previous 5 years, or any uncured cancer diagnosed more than 5 years ago (with clinical evidence of relapse in the previous 5 years). All included patients provided written, informed consent. The study was approved by the relevant Ethics Committees and conducted in accordance with ICH GCP, the Declaration of Helsinki 2008 and any applicable local requirements.

Patients were asked to complete three validated QoL questionnaires: European Organisation for Research and Treatment of Cancer Quality of Life Questionnaire-Prostate Cancer 25 (EORTC QLQ-PR25); EORTC Quality of Life Questionnaire—Cancer 30 (EORTC QLQ-C30); and EuroQoL-5D (EQ-5D) [12–14]. Anxiety and depression were also assessed, using the Hospital Anxiety and Depression Scale (HADS) questionnaire. In addition, the Work Productivity Assessment Index (WPAI) questionnaire was used to assess effect of diagnosis and treatment on work productivity and activity. All questionnaires were completed at baseline (within 2 months of diagnosis and before any prostate cancer treatment), and at 3 months and 12 months after starting prostate cancer treatment. Questionnaires were completed in the clinic in a quiet room, away from any influence of healthcare professionals. In extenuating circumstances, questionnaires could be completed at home and returned to the clinic by post. Missing values were not imputed.

The primary study endpoint was the change in QLQ-PR25 urinary symptoms subscale score from baseline to the assessment at 3 months after the start of prostate cancer treatment. Secondary endpoints were changes in other QLQ-PR25 subscale scores from baseline to the 3-month assessment; changes in all QLQ-PR25 subscale scores from baseline to the assessment after 12 months' treatment; changes in QLQ-C30 scores from baseline to the 3- and 12-month assessments; difference from normative data (based on the UK general population) in baseline EQ-5D and HADS scores; changes in EQ-5D scores from baseline to the 3- and 12-month assessments; changes in HADS scores from baseline to the 3- and 12-month assessments; and cost assessment for prostate cancer subjects based on type of treatment administered, resource utilisation (visits/treatment), and indirect costs captured using the WPAI.

A total of 134 patients per country were needed in order to have 90% power to detect a difference of 4.9 from baseline to 3 months in QLQ-PR25 urinary symptoms subscale scores. The Full Analyses Set (FAS) consisted of all patients who completed the baseline and the 3-month QLQ-PR25 questionnaire and was used for the primary outcome analysis. The Baseline Analyses Set (BAS) consisted of all patients who completed the baseline EQ-5D and HADS questionnaire. The primary endpoint was assessed for the FAS population (observed cases) using a repeated measures analysis of variance with the following covariates: age, centre, initial treatment received, Gleason score, T-stage, PSA test result, education status, and whether the subject had a progressive benign prostatic hyperplasia (BPH) diagnosis (defined by acute urinary retention [AUR]/BPH surgery). For the study endpoints, two-sided 95% confidence intervals for the adjusted mean changes from baseline in the questionnaire scores were calculated along with *P* values from a two-sided significance test that the mean change from baseline was zero. A significance level of 0.05 was used for the primary endpoint.

## 3. Results

### 3.1. Patients

A total of 672 patients were enrolled, of whom 603 completed the baseline EQ-5D and HADS questionnaires (BAS population; 86 patients (18 centres) in France, 132 (20 centres) in Germany, 131 each in Italy (9 centres) and in Spain (11 centres), and 123 (9 centres) in Sweden) and 404 completed the baseline and 3-month QLQ-PR25 questionnaires (FAS population); 326 patients completed the 12-month QLQ-PR25 questionnaire. Information on why patients did not complete the questionnaire was not collected. For the BAS population, the median study duration was 398 days (range: 1 to 787 days). Demographic and baseline characteristics are summarised in Table 1. Mean (SD) age was 65.0 (5.7) years and 400 (66%) men had Gleason score ≤6 prostate cancer. Mean (SD) PSA level at baseline was 7.2 (3.4) ng/mL. Demographic characteristics were largely similar in subjects who did and those who did not complete the questionnaires.

The most frequently used initial treatment for prostate cancer was radical prostatectomy (71% of patients). Other treatments were external beam radiotherapy (9%), brachytherapy (3%), combined hormonal therapy/radiotherapy (2%), hormonal therapy alone (1%), and radical prostatectomy followed by salvage radiotherapy (<1%). Ten percent of patients were subjected to active surveillance/watchful waiting, and 2% received other treatment. A total of 176 patients were receiving concomitant medications for genitourinary conditions, the most common of which were alprostadil (7%), tadalafil (7%), bicalutamide (6%), and tamsulosin (6%).

### 3.2. QLQ-PR25

QLQ-PR25 urinary symptoms subscale score was significantly increased at 3 months (*P* < 0.001), indicating that urinary symptoms worsened after treatment (Table 2). The score was lower at 12 months than at 3 months, but it was still significantly higher than at baseline (*P* < 0.001). Of the covariates assessed, age (*P* < 0.0001) and centre (*P* = 0.0003) were significant in relation to this subscale score. Analysis of QLQ-PR25 urinary symptoms subscale score was also performed according to initial treatment of prostate cancer. Among the 71% of subjects who underwent a radical prostatectomy, statistically significantly increases in QLQ-PR25 urinary symptoms subscale scores were seen after treatment (*P* < 0.001), similar to the results for the overall population. For subjects who underwent external radiotherapy, a statistically significant increase in QLQ-PR25 urinary symptoms subscale score was seen at month 3 (*P* = 0.047) but not at month 12 (*P* = 0.103). In patients managed with an active surveillance approach, symptoms worsened but this was not statistically significant at either time point. In country-specific analyses, urinary symptoms significantly worsened in all five countries at 3 months, and in Germany and France at 12 months.

Other QLQ-PR25 subscale score endpoints are reported in Table 3. Hormonal treatment-related symptoms, sexual functioning, and sexual activity scores significantly worsened at 3 and 12 months (all *P* < 0.001). Incontinence aid problems score increased, but this was only significant at 3 months (*P* = 0.003; *P* = 0.075 at 12 months). There was no significant change in bowel symptoms score at either time point.

### 3.3. QLQ-C30

For the QLQ-C30 questionnaire, global health status/QoL score significantly decreased at month 3 but was not different from baseline by month 12. Scales for physical, role and social functioning, and fatigue, showed significant deterioration at 3 and 12 months. No significant change was observed in cognitive functioning, while emotional functioning significantly improved. Pain score was significantly worse compared with baseline at month 3 but not at month 12. Nausea and vomiting score was largely unaffected (Table 4).

### 3.4. EQ-5D, HADS, and WPAI

There was no significant change from baseline in EQ-5D scores at 3 and 12 months following treatment for prostate cancer (data not shown). Compared with age-matched normative data (UK general population), the health status (EQ-5D) of the study population was similar to the general population at baseline and month 12, but significantly worse 3 months after starting treatment.

Anxiety score (HADS anxiety subscale) was significantly reduced (*P* < 0.001) from baseline (5.3) at both 3 (4.4) and 12 (4.3) months following the start of treatment. The largest improvement at both time points was in the group of patients (*n* = 12) whose initial management was with watchful waiting. However, depression score (HADS depression subscale) did not change significantly. Compared with age-matched normative data (UK general population), anxiety was significantly lower (*P* < 0.001) in the study population at baseline, 3, and 12 months, while depression was similar at all three time points.

Approximately 25% of patients were employed at the time of entering the study. Fewer patients remained in work after they received a prostate cancer treatment (19% at month 3 and 16% at month 12). On average, 32 working hours in the previous week were reported at baseline; these hours slightly decreased following initial treatment. The average missed working hours due to prostate cancer in the previous week was 6 hours at baseline and month 3, and 2 hours at month 12. Based on the overall WPAI scores, diagnosis and treatment of prostate cancer had no impact on working productivity and on regular daily activities over the course of the study.

Medical costs including resource utilisation associated with prostate cancer diagnosis and/or treatment were analysed. Of 603 subjects in the BAS population, 96% (*n* = 578) had a consultation(s) with a healthcare professional for management of their prostate cancer. The primary reason for the consultation was related to diagnosis and/or monitoring of their prostate cancer. Ninety-seven percent of subjects (*n* = 586) had at least one type of procedure related to their prostate cancer management. Procedures were related to diagnosis and/or monitoring of prostate cancer in 89% of patients, with PSA testing and biopsy being the most frequently performed. Procedures were related to prostate cancer treatment in 83% of subjects, with more than half of these (*n* = 285) undergoing a radical prostatectomy.

## 4. Discussion

In this observational study, the majority of patients recruited were due to receive treatment of curative intent such as radical prostatectomy, external radiotherapy, brachytherapy, and hormone therapy, despite having tumours of low-to-moderate risk. For example, almost three-quarters of patients (71%) underwent radical prostatectomy as primary treatment. Overall, treatment of prostate cancer had negative effects on all six domains of the prostate cancer-specific QLQ-PR25 questionnaire. Urinary symptoms were generally worse after 3 months and although they improved after 12 months, they remained significantly worse than at baseline. Incontinence aid problems were also worse after 3 months than after 12 months (only significant versus baseline at month 3). Unlike urinary symptoms, sexual functioning did not tend to improve after one year compared with 3 months, suggesting that the impact of treatment on sexual function may be longer-lasting and more profound compared with the effect on urinary symptoms. On the other hand, sexual activity score did show a slight improvement after 12 months compared with 3 months, although it remained significantly lower compared with baseline. Bowel symptoms scores worsened, particularly among patients whose initial treatment included radiotherapy (data not shown), although overall the change from baseline was not statistically significant. Worsening of urinary symptoms among patients managed with active surveillance may suggest an age-related natural decline, although age was taken into account in our analyses as a covariate.

The primary endpoint for the study was the change in QLQ-PR25 urinary symptoms subscale score from baseline to the assessment at 3 months after the start of prostate cancer treatment. For some prostate cancer interventions (e.g., radiation therapy), the adverse effects are not immediately evident; the 3-month time point was therefore selected on the basis that this would enable the adverse impact of most interventions to become apparent. The urinary symptoms subscale score was chosen in order to have a primary endpoint that was common across all prostate cancer interventions. The number of evaluable patients was less than planned, which had the potential to adversely affect power. However, despite this, statistically significant differences were observed for the primary endpoint, both overall and in each individual country.

Using the more general cancer QoL QLQ-C30 questionnaire, physical, role, and social functioning significantly worsened following treatment, as did fatigue and pain symptoms. In contrast, emotional functioning improved after treatment. Changes in QLQ-PR25 and C30 questionnaires were not strongly correlated with health status (as assessed by EQ-5D scores), which did not significantly change from baseline. One possible explanation for this lack of correlation is that the EQ-5D assessment may not have been sensitive enough to reflect the changes in health-related QoL that occur following interventions for prostate cancer.

Patients' anxiety and depression levels were relatively unaffected at the time of diagnosis, as mean scores were within the normal range of the HADS questionnaire. These scores might have been expected to deteriorate over time, as other studies have shown a high incidence of anxiety, depression, and distress [7, 15]. However, depression remained largely unchanged while anxiety actually lessened after treatment. This is consistent with the improvement in emotional functioning as measured by the subscale of the QLQ-C30 questionnaire. It is possible that therapeutic intervention might allay patient fears and so reduce anxiety. Education of patients, psychological support from healthcare providers and caregivers, and the use of anxiolytic medications might also have been factors in reducing anxiety and improving emotional functioning.

The majority of patients (75%) were not working at study entry, thereby limiting the data on the impact of prostate cancer on work productivity. However, for those subjects who did work our data suggest that any impact on work productivity may be transitory, and improves over time, following prostate cancer treatment.

Our study primarily examined the burden of low-to-moderate risk prostate cancer from the perspective of the disease state. Nevertheless, our findings are generally in line with those from other studies that have assessed the reported impact of prostate cancer treatment on various aspects of physical functioning. Radical prostatectomy, radiotherapy, hormonal therapy, and watchful waiting have all been shown to have a negative impact on sexual function, urinary function, and bowel function of patients treated for prostate cancer [6, 8, 9, 15–18]. Most recently, an analysis of 1655 men from the Prostate Cancer Outcomes Study compared long-term urinary, bowel, and sexual function after radical prostatectomy or external-beam radiation therapy for localised prostate cancer [19]. This study showed that these treatments resulted in declines in all functional domains during 15 years of follow-up. The absence of any significant effect on bowel symptoms in our study may be explained by the fact that only approximately 10% of patients received external radiotherapy, the treatment most associated with bowel toxicity [20].

A potential limitation of our study is that the results rely on patient recall after 3 months and after 12 months. Our study may also have benefited from a longer follow-up period than 12 months. In addition, because of the observational nature of the study, there may be differences in factors such as age, Gleason score, and tumour stage according to initial treatment received; this may restrict comparison of the data according to initial treatment type, and also prevented any further subgroup analyses according to initial treatment received. Another possible limitation is the high proportion of patients treated with radical prostatectomy, which may limit the external validity of our findings. However, it is important to note that interventions were not restricted in the study protocol, with treatment decisions left to the physician/patient based on individual patient circumstances. In this respect, our study population reflects real-life clinical practice. Further, the proportion of patients treated by surgery in this study is consistent with other published data that show radical prostatectomy is the most common treatment in men with low-to-moderate risk prostate cancer [4, 21].

In the present study, statistically significant differences compared with baseline were demonstrated for several of the QoL subscales assessed; however, this does not necessarily translate to clinically important effects. There is currently no definition available as to what would represent a clinically meaningful change in QLQ-PR25 scores, although some information is available on the minimal clinically important difference in QLQ-C30 scores [22]. These data suggest that changes in the range of 5–10% (or 5–10 points in the present study) may be considered clinically significant.

QoL considerations are important in helping guide treatment decision-making in patients with prostate cancer, especially those at low-to-moderate risk of progression. The majority of these patients have a favourable prognosis and are not destined to die of their disease even in the absence of treatment. However, overtreatment of indolent disease may be a problem given that the various treatment options can have a significant negative impact on a patient's health status and QoL. This study showed that, across France, Germany, Italy, Spain, and Sweden, low-to-moderate risk prostate cancer is usually treated with radical therapies. This treatment strategy had a negative impact on various dimensions of QoL in these patients during the one-year observation period. Prostate cancer treatment was associated with a decrease in urinary and sexual functioning and increase in hormonal treatment-related symptoms. With some exceptions, the impact on treatment-related functioning scales and symptoms tended to be higher at month 3 and to have some degree of improvement at month 12. Treatment of prostate cancer had minimal effects on depression and anxiety, and a limited impact on productivity among active workers.

In conclusion, low-to-moderate risk prostate cancer may have a substantial effect on the QoL of affected patients within one year following treatment. Our study provides further supportive information on the QoL impact of treatments for low-to-moderate risk prostate cancer and will help physicians to tailor discussions with patients and guide decision making for disease management, particularly with regard to the primary treatment chosen.

## Figures and Tables

**Table 1:** Baseline demographics and clinical characteristics (BAS population).

	Overall *N* = 603
Demographic characteristics	
Age (yrs), *n*	603
Mean ± SD	65.0 ± 5.73
Median (range)	66.0 (50–75)
Education, *n* (%)	
Less than high school	242 (40%)
High school	179 (30%)
Some college/university	69 (11%)
College/university graduate	46 (8%)
Post graduate (M.S., Ph.D.)	15 (2%)
Do not care to answer	52 (9%)
Family history of prostate cancer, *n* (%)	104 (17%)
Father	60 (10%)
Brother	38 (6%)
Grandfather	13 (2%)
Uncle	13 (2%)
Son	0

Disease characteristics	
Total Gleason score, *n* (%)	603
≤6	400 (66%)
7	203 (34%)
PSA^a^ (ng/mL), *n*	603
Mean ± SD	7.207 ± 3.4348
Median (range)	6.35 (0.01–19.30)
≤10 ng/mL, *n* (%)	505 (84%)
11–20 ng/mL, *n* (%)	98 (16%)
Clinical staging, *n* (%)	603
T1a	8 (1%)
T1b	5 (<1%)
T1c	355 (59%)
T2a	123 (20%)
T2b	112 (19%)
Clinical BPH^b^ diagnosis, *n* (%)	603
Yes	225 (37%)
Progressive BPH diagnosis^c^, *n* (%)	225
Yes	34 (15%)

^a^PSA: prostate-specific antigen.

^b^BPH: benign prostatic hyperplasia.

^c^Defined by acute urinary retention (AUR) and BPH-related surgery.

**Table 2:** Change from baseline in QLQ-PR25 urinary symptoms subscale score (FAS population, observed cases).

QLQ-PR25 urinary symptoms^a,b^	*n*	Adjusted mean ± SE *N* = 404	Adjusted mean change from baseline (95% CI) *N* = 404	*P* value for change from baseline *N* = 404
Baseline	401	14.7 ± 3.01		
Month 3	403	24.1 ± 3.05	9.36 (7.47, 11.25)	<0.001
Month 12	326	19.2 ± 3.03	4.43 (2.70, 6.16)	<0.001

^a^Covariates included terms for age, centre, initial treatment received, Gleason score, T-stage, PSA test result, education status, and progressive BPH diagnosis.

^b^Scale range is 0 to 100. Higher scores indicate worse symptoms.

**Table 3:** Change from Baseline in other QLQ-PR25 Subscale Scores (FAS Population, Observed Cases).

QLQ-PR25 subset^a^	*n*	Adjusted mean ± SE *N* = 404	Adjusted mean change from baseline (95% CI) *N* = 404	*P* value for change from baseline *N* = 404
Incontinence aid poblems^c^				
Baseline	49	−0.9 ± 11.18		
Month 3	202	15.0 ± 10.65	15.81 (5.76, 25.85)	0.003
Month 12	124	8.8 ± 10.83	9.63 (−1.01, 20.27)	0.075
Bowel symptoms^c^				
Baseline	399	6.2 ± 1.53		
Month 3	399	6.8 ± 1.53	0.60 (−0.24, 1.45)	0.159
Month 12	318	7.0 ± 1.54	0.84 (−0.14, 1.81)	0.093
Treatment-related symptoms^c^				
Baseline	371	7.6 ± 1.82		
Month 3	376	13.0 ± 1.85	5.42 (4.40, 6.43)	<0.001
Month 12	306	12.7 ± 1.85	5.11 (4.00, 6.21)	<0.001
Sexual Functioning^b^				
Baseline	293	79.7 ± 4.62		
Month 3	221	53.1 ± 4.67	−26.54 (−30.57, −22.50)	<0.001
Month 12	194	52.0 ± 4.67	−27.67 (−31.43, −23.91)	<0.001
Sexual activity^b^				
Baseline	397	33.2 ± 4.75		
Month 3	401	23.0 ± 4.73	−10.29 (−12.99, −7.59)	<0.001
Month 12	323	25.8 ± 4.77	−7.39 (−10.34, −4.44)	<0.001

^a^Covariates included terms for age, centre, initial treatment received, Gleason Score, T-stage, PSA test result, education status and progressive BPH diagnosis.

^b^Range for each scale is 0 to 100. Higher scores indicate better functioning.

^c^Range for each scale is 0 to 100. Higher scores indicate worse symptoms/more problems.

**Table 4:** Change from baseline in QLQ-C30 scales (FAS population, observed cases).

QLQ-C30 subset^a^	*n*	Adjusted mean ± SE *N* = 404	Adjusted mean change from baseline (95% CI) *N* = 404	*P* value for change from baseline *N* = 404
Global health status/QoL scale^b^				
Baseline	400	74.2 ± 3.81		
Month 3	401	71.0 ± 3.81	−3.19 (−5.26, −1.12)	0.003
Month 12	323	74.2 ± 3.82	−0.02 (−2.25, 2.22)	0.987
Physical functioning scale^b^				
Baseline	397	90.3 ± 2.24		
Month 3	394	86.5 ± 2.29	−3.81 (−5.03, −2.59)	<0.001
Month 12	322	88.3 ± 2.29	−1.95 (−3.18, −0.72)	0.002
Role functioning scale^ b^				
Baseline	400	88.2 ± 3.17		
Month 3	400	79.4 ± 3.27	−8.81 (−11.05, −6.58)	<0.001
Month 12	324	84.5 ± 3.24	−3.77 (−5.65, −1.88)	<0.001
Emotional functioning scale^b^				
Baseline	396	80.1 ± 3.88		
Month 3	401	83.3 ± 3.86	3.20 (1.26, 5.13)	0.001
Month 12	323	86.3 ± 3.84	6.26 (4.37, 8.14)	<0.001
Cognitive functioning scale^b^				
Baseline	302	82.5 ± 4.88		
Month 3	305	81.3 ± 4.90	−1.21 (−2.92, 0.49)	0.162
Month 12	245	81.0 ± 4.89	−1.51 (−3.15, 0.13)	0.071
Social functioning scale^b^				
Baseline	399	89.2 ± 3.40		
Month 3	400	82.3 ± 3.45	−6.89 (−8.92, −4.85)	<0.001
Month 12	323	85.4 ± 3.43	−3.83 (−5.74, −1.92)	<0.001
Fatigue scale^c^				
Baseline	399	12.8 ± 3.47		
Month 3	399	18.0 ± 3.50	5.22 (3.53, 6.90)	<0.001
Month 12	322	15.5 ± 3.50	2.78 (1.26, 4.30)	<0.001
Nausea and vomiting scale^c^				
Baseline	402	4.0 ± 1.31		
Month 3	401	4.0 ± 1.29	0.06 (−0.79, 0.92)	0.882
Month 12	325	4.2 ± 1.29	0.20 (−0.67, 1.07)	0.652
Pain scale^c^				
Baseline	399	17.9 ± 3.32		
Month 3	400	21.2 ± 3.36	3.36 (1.26, 5.47)	0.002
Month 12	326	17.5 ± 3.32	−0.38 (−2.06, 1.29)	0.652

^a^Covariates included terms for age, centre, initial treatment received, Gleason Score, T-stage, PSA test result, education status, and progressive BPH diagnosis. Family history of breast cancer was also included for role functioning, emotional functioning, and pain sclae. Ethnicity was also included for cognitive functioning.

^b^Range for each scale is 0 to 100. Higher scores indicate better functioning.

^c^Range for each scale is 0 to 100. Higher scores indicate worse symptoms.
